# Shotgun metagenomics and metatranscriptomics of soil microbial communities under monoculture and polyculture cover crops

**DOI:** 10.1128/mra.00309-26

**Published:** 2026-05-28

**Authors:** Nichole Giani, Jojy John, Barbara Campbell

**Affiliations:** 1 Department of Biological Sciences, Clemson University 170362, Clemson, South Carolina, USA; Indiana University Bloomington, Bloomington, Indiana, USA

**Keywords:** rhizosphere microbiome, cover crops, agroecosystems, metagenomics, metatranscriptomics, functional redundancy, monoculture and polyculture

## Abstract

Here, we present 30 metagenomes, 21 metatranscriptomes, and 334 metagenome-assembled genomes collected from soils under different cover crop species. This data set will be useful for studying microbial interactions, especially functional redundancy, with relevance to agricultural management and sustainability.

## ANNOUNCEMENT

Agricultural soils harbor diverse microbial communities that regulate carbon and nutrient cycling, plant nutrient availability, and ecosystem stability (1, 2). Cover crops are widely used to improve soil health, suppress weeds, and enhance nutrient retention; however, genome-resolved studies of rhizosphere microbial communities under monoculture and polyculture systems remain limited. Here, we present metagenomes, metatranscriptomes, and metagenome-assembled genomes (MAGs) from rhizosphere soils across cover crop treatments, providing a resource to examine microbial functional potential, activity, and niche differentiation in agroecosystems.

Rhizosphere soils were collected during the 2023–2024 growing season from a replicated field experiment at Simpson’s Farm in Pendleton, South Carolina, USA (34.655245° N, 82.735083° W). Treatments included monocultures of field pea (*Pisum sativum*), forage radish (*Raphanus sativus*), and cereal rye (*Secale cereale*); a three-species mixture (field pea, oats [*Avena sativa*], forage radish); a five-species mixture (field pea, red clover [*Trifolium pratense*], crimson clover [*Trifolium incarnatum*], cereal rye, canola [*Brassica napus*]); and a no-cover control. Soils (0 cm–10 cm) were collected at cover crop termination (March 2024) using three cores per plot (composited to make a representative sample), composited, flash-frozen, and stored at −80°C. Nucleic acids were co-extracted from 500 mg of soil using the modified phenol–chloroform protocol by Griffiths et al. (3), purified with the Zymo Clean & Concentrator-5 kit (Zymo Research, Irvine, CA, USA), and quantified with Qubit dsDNA HS and RNA HS assays (Thermo Fisher Scientific, Waltham, MA, USA). Metagenomic libraries were prepared using the Illumina DNA Prep kit, and RNA-seq libraries were prepared using the Stranded Total RNA Prep with Ribo-Zero Plus kit with unique dual indexes (Illumina, San Diego, CA, USA). Sequencing was performed on an Illumina NextSeq 2000 platform (300-cycle P4 XLEAP chemistry), generating paired-end reads.

Raw metagenomic reads were quality filtered and adapter trimmed using Trimmomatic (0.39) and fastp (0.23.2), with quality assessed by FastQC (0.12.1), yielding 122.5 million paired-end reads (4–6). Reads were combined into two large paired-end files (1.36 billion reads total) and co-assembled using MEGAHIT (v.1.2.9) (7). Reads were mapped to the assembly with Bowtie2 (v.2.5.4) to generate coverage profiles (8). MAGs were recovered using SemiBin2 (v.2.2.1) with sequence composition and multi-sample coverage features (9). Genome quality was assessed with CheckM2 (v.1.1.0) (10), retaining bins with ≥70% completeness and ≤10% contamination, and dereplicated using dRep (v.3.4.2) (11). Individual metagenomic and metatranscriptomic data are provided in Table 1. All tools were run with default parameters unless otherwise noted.

**TABLE 1:** Accession numbers and characteristics of metagenomes and metatranscriptomes from the different rhizosphere samples

BioSample accession no.	SRA accession no.	LibraryID	Nucleic acid	Cover_Crop	Plot no.	Replicate	Paired-end reads
SAMN54986593	SRR37058681	5spp_5_R1_S8	DNA	5spp	5	R1	58,245,055
SAMN54986591	SRR37058683	5spp_16_R1_S6	DNA	5spp	16	R1	75,234,172
SAMN54986590	SRR37058684	3spp_20_R2	DNA	3spp	20	R2	66,992,384
SAMN54986590	SRR37058684	3spp_20_R2_RNA	RNA	3spp	20	R2	36,319,089
SAMN54986589	SRR37058685	3spp_8_R2_S5	DNA	3spp	8	R2	61,728,244
SAMN54986615	SRR37058686	Rye_21_R2	DNA	Rye	21	R2	80,221,652
SAMN54986615	SRR37058686	Rye_21_R2_RNA	RNA	Rye	21	R2	17,952,999
SAMN54986614	SRR37058687	Rye_14_R2_S29	DNA	Rye	14	R2	82,506,321
SAMN54986613	SRR37058688	Rye_14_R1_S28	DNA	Rye	14	R1	46,340,794
SAMN54986609	SRR37058692	Radish_24_R2_S24	DNA	Radish	24	R2	37,705,930
SAMN54986608	SRR37058693	Radish_24_R1_S23	DNA	Radish	24	R1	51,646,690
SAMN54986605	SRR37058697	Pea_2_R1_S19	DNA	Pea	2	R1	49,829,987
SAMN54986601	SRR37058701	Pea_12_R2_S16	DNA	Pea	12	R2	36,826,267
SAMN54986600	SRR37058702	Control_6_R2_S15	DNA	Control	6	R2	65,790,239
SAMN54986596	SRR37058706	Control_15_R1_S11	DNA	Control	15	R1	48,012,075
SAMN54986586	SRR37058708	3spp_18_R1_S1	DNA	3spp	18	R1	94,762,133
SAMN54986602	SRR37059088	Pea_17_R1_S17	DNA	Pea	17	R1	117,175,480
SAMN54986602	SRR37059088	Pea_17_R1_S17_RNA	RNA	Pea	17	R1	34,331,847
SAMN54986599	SRR37059089	Control_6_R1_S14	DNA	Control	6	R1	41,996,131
SAMN54986599	SRR37059089	Control_6_R1_S14_RNA	RNA	Control	6	R1	43,393,214
SAMN54986598	SRR37059090	Control_23_R1_S13	DNA	Control	23	R1	68,763,900
SAMN54986598	SRR37059090	Control_23_R1_S13_RNA	RNA	Control	23	R1	36,456,182
SAMN54986597	SRR37059091	Control_15_R2_S12	DNA	Control	15	R2	50,878,389
SAMN54986597	SRR37059091	Control_15_R2_S12_RNA	RNA	Control	15	R2	41,118,002
SAMN54986595	SRR37059092	5spp_9_R1_S10	DNA	5spp	9	R1	142,294,723
SAMN54986595	SRR37059092	5spp_9_R1_S10_RNA	RNA	5spp	9	R1	28,546,992
SAMN54986594	SRR37059093	5spp_5_R2_S9	DNA	5spp	5	R2	48,451,781
SAMN54986594	SRR37059093	5spp_5_R2_S9_RNA	RNA	5spp	5	R2	40,947,064
SAMN54986592	SRR37059094	5spp_16_R2_S7	DNA	5spp	16	R2	41,683,750
SAMN54986592	SRR37059094	5spp_16_R2_S7_RNA	RNA	5spp	16	R2	38,255,500
SAMN54986612	SRR37059097	Rye_10_R2_S27	DNA	Rye	10	R2	69,872,625
SAMN54986612	SRR37059097	Rye_10_R2_S27_RNA	RNA	Rye	10	R2	47,355,691
SAMN54986611	SRR37059098	Rye_10_R1_S26	DNA	Rye	10	R1	41,106,620
SAMN54986611	SRR37059098	Rye_10_R1_S26_RNA	RNA	Rye	10	R1	36,116,822
SAMN54986610	SRR37059099	Radish_4_R1_S25	DNA	Radish	4	R1	52,116,721
SAMN54986610	SRR37059099	Radish_4_R1_S25_RNA	RNA	Radish	4	R1	22,802,888
SAMN54986607	SRR37059100	Radish_11_R2_S22	DNA	Radish	11	R2	64,350,882
SAMN54986607	SRR37059100	Radish_11_R2_S22_RNA	RNA	Radish	11	R2	35,521,956
SAMN54986606	SRR37059101	Radish_11_R1_S21	DNA	Radish	11	R1	63,350,277
SAMN54986606	SRR37059101	Radish_11_R1_S21_RNA	RNA	Radish	11	R1	44,042,435
SAMN54986604	SRR37059102	Pea_22_R2_S20	DNA	Pea	22	R2	171,445,257
SAMN54986604	SRR37059102	Pea_22_R2_S20_RNA	RNA	Pea	22	R2	26,425,567
SAMN54986603	SRR37059103	Pea_17_R2_S18	DNA	Pea	17	R2	34,801,085
SAMN54986603	SRR37059103	Pea_17_R2_S18_RNA	RNA	Pea	17	R2	24,551,533
SAMN54986588	SRR37059104	3spp_3_R1_S4	DNA	3spp	3	R1	60,773,870
SAMN54986588	SRR37059104	3spp_3_R1_S4_RNA	RNA	3spp	3	R1	35,293,296
SAMN54986587	SRR37059105	3spp_20_R1_S2	DNA	3spp	20	R1	82,135,319
SAMN54986587	SRR37059105	3spp_20_R1_S2_RNA	RNA	3spp	20	R1	31,772,276
SAMN55017259	SRR37076429	Radish_13_13_S11_RNA	RNA	Radish	13	R2	40,486,120
SAMN55017258	SRR37076430	3spp_3_12_S10_RNA	RNA	3spp	3	R2	54,622,043
SAMN55017257	SRR37076431	3spp_20_18_S15_RNA	RNA	3spp	20	R2	34,914,175

Taxonomic classification using GTDB-Tk (v.2.6.1) (12) identified 338 bacterial and 10 archaeal MAGs. Dominant bacterial classes included Thermoleophilia (*n* = 50), Alphaproteobacteria (*n* = 54), Terriglobia (*n* = 43), Gemmatimonadetes (*n* = 30), Vicinamibacteria (*n* = 25), Gammaproteobacteria (*n* = 22), and Actinomycetes (*n* = 19), with additional representation from Acidimicrobiia, Nitrospiria, Verrucomicrobiae, Chloroflexia, Ktedonobacteria, and Bacteroidia. Archaeal MAGs were primarily classified as Nitrososphaeria.

## Data Availability

All metagenomes, metatranscriptomes, and MAGs are available through NCBI under BioProject PRJNA1416991. Raw metagenomic and metatranscriptomic BioSample accessions are SAMN54986586–SAMN54986615 and SAMN55017257–SAMN55017259, respectively. MAG BioSample accessions are SAMN54997129–SAMN54997483. Table 2 (MAG statistics) is uploaded to figshare (https://doi.org/10.6084/m9.figshare.31690180).
